# Single-stage multidisciplinary surgical repair of a secondary aortoesophageal fistula: A technical case report with 3-year uneventful follow-up

**DOI:** 10.1016/j.xjtc.2026.102417

**Published:** 2026-05-15

**Authors:** Adam Zeyara, Firas Al Saedi, Torbjörn Fransson, Shahab Nozohoor, Jonas Ahl, Per Wierup, Jan Johansson

**Affiliations:** a Department of Upper GI Surgery, Skåne University Hospital, Lund, Sweden; b Department of General Surgery, Skåne University Hospital, Malmö, Sweden; c Department of Vascular Surgery, Skåne University Hospital, Malmö, Sweden; d Department of Thoracic and Cardiovascular Surgery, Skåne University Hospital, Lund, Sweden; e Department of Infectious Diseases, Skåne University Hospital, Malmö, Sweden

**Keywords:** aortoesophageal fistula, surgical repair, single-stage, long-term follow-up

## Abstract

**Objective:**

Secondary aortoesophageal fistula (AEF) after thoracic endovascular aortic repair (TEVAR) is a rare but life-threatening complication. Definitive treatment guidelines are lacking, and surgical management has traditionally relied on staged procedures. Reports of successful single-stage repair with long-term follow-up remain scarce. This case report aims to describe the workup, surgical strategy, and long-term outcomes of a single-stage multidisciplinary repair of secondary AEF after TEVAR.

**Methods:**

We report the case of a 75-year-old man who presented with recurrent infectious episodes 9 years after TEVAR. A secondary AEF was diagnosed in May 2022. After the failure of conservative treatment (ie, antibiotic therapy) over approximately 1 year, a multidisciplinary team recommended definitive surgery. In April 2023, a single-stage repair was performed, comprising complete resection of the infected stent graft, in situ replacement with an antibiotic-soaked prosthesis, primary esophageal repair, and interposition of a pedicled latissimus dorsi muscle flap.

**Results:**

The patient was discharged on postoperative day 30. Three-year follow-up demonstrated sustained remission with normalized inflammatory markers and no evidence of graft reinfection.

**Conclusions:**

In this case report, a single-stage multidisciplinary surgical repair of secondary AEF after TEVAR yielded durable infection control and favorable long-term outcomes. Careful patient selection within a multidisciplinary team, meticulous surgical planning, and close postoperative infectious disease follow-up are essential. Three-year follow-up demonstrated sustained remission with normalized inflammatory markers and no evidence of graft reinfection.

**Figure:**
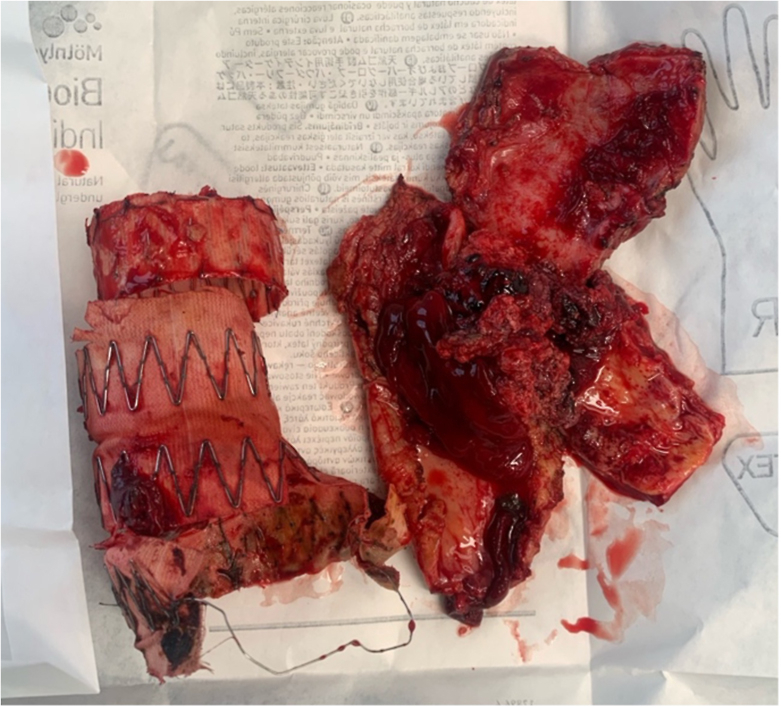
The picture depicts the extracted infected stent graft with parts of the aneurysmal sac.

**Central Message**

Single-stage resection and reconstruction for secondary aortoesophageal fistula after TEVAR achieved durable infection control and uneventful 3-year follow-up in a multidisciplinary setting.

**Perspective**

Secondary aortoesophageal fistula after TEVAR is a rare, life-threatening complication without clear management guidelines. This case shows that in selected patients, single-stage graft explantation, in situ reconstruction, and primary esophageal repair with muscle flap interposition can provide definitive infection control and durable 3-year outcomes through multidisciplinary care.

Secondary aortoesophageal fistulas (AEFs) most commonly occur after open or endovascular surgical intervention of the aorta. After aortic stent graft placement, the underlying mechanism is thought to involve mechanical irritation of surrounding tissues by the residual aneurysmal sac with or without possible residual pressurization (endoleak). Cardiac pulsation and esophageal peristalsis, combined with local inflammation, may lead to progressive erosion of the aneurysmal and esophageal walls, ultimately resulting in fistula formation and chronic infection.^1^^,^^2^

Patients with AEF typically present with gastrointestinal bleeding, which may rapidly progress to a life-threatening condition.^3^ Another diagnostic presentation includes recurrent or persistent infections with limited response to antibiotic therapy.^4^ Secondary AEFs may occur as early as 2 weeks after aortic surgery or more than 10 years postoperatively.^2^ Diagnosis is generally established through a combination of computed tomography (CT) imaging and esophagoscopy.^4^

Management strategies depend on the patient’s clinical presentation and may include conservative treatment, endovascular restenting, surgical treatment, or palliative care. Because of the rarity of this condition, definitive treatment guidelines for AEF are lacking, and management is typically tailored to the individual patient.^1^^,^^4^ Thoracic endovascular aortic repair (TEVAR) is often considered the first-line intervention to control life-threatening hemorrhage, which is the most common presenting symptom.^5^ TEVAR may also serve as a bridge to definitive surgical treatment in cases in which graft infection represents the primary pathology.^6^ A systematic review has demonstrated that surgery is associated with significantly greater mortality rates compared with endovascular approaches; however, endovascular methods carry a significant risk of persistent chronic infections.^7^ Thus, definitive surgical treatment with graft explantation seems to be the preferable management in patients who are fit for surgery.^6^, ^7^, ^8^

Traditionally, surgical treatment in this context has often been performed as a staged procedure, commonly involving 2 or even 3 operative stages. Most involve initial esophageal diversion or esophagectomy, with immediate or delayed reconstruction, followed by definitive vascular repair. This approach is intended to allow full recovery before gastrointestinal reconstruction, which is typically performed later using a separate anatomical compartment, such as the retrosternal route.^8^, ^9^, ^10^, ^11^ Although single-stage procedures have shown promise, the supporting literature remains very limited, making every contribution to this evidence base valuable, particularly data with long-term follow-up.^12^^,^^13^ We describe a successful single-stage surgical approach for secondary AEF, with the patient discharged within 30 days postoperatively and 3 years of uneventful clinical follow-up.

## Case Presentation

Written informed consent was obtained from the patient for publication of this case report and any accompanying images. A copy of the written consent is available for review by the Editor-in-Chief of this *Journal* on request. All data are available upon reasonable request.

A 75-year-old male patient with a 60-mm asymptomatic aneurysm of the proximal descending thoracic aorta underwent TEVAR (Cook TX2 42/126) in 2013. The stent graft was positioned just distally to the subclavian ostium and extended into the abdomen above the celiac trunk with a GORE TAG 42/200. The initial postoperative course was uneventful. However, 2 years later, a small type II endoleak was detected but with no expansion over time. Conservative management with continued CT surveillance was adopted. The patient remained asymptomatic and functionally independent for approximately 9 years.

**Figure 1:**
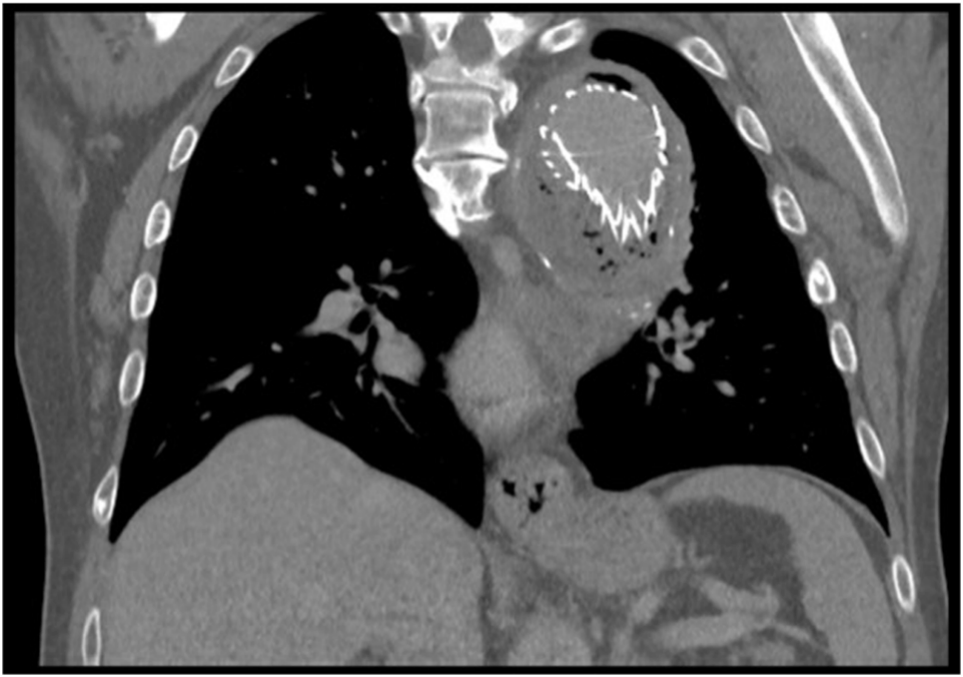
Computed tomography of the infected aneurysm sac and stent graft at the time of diagnosis.

**Figure 2:**
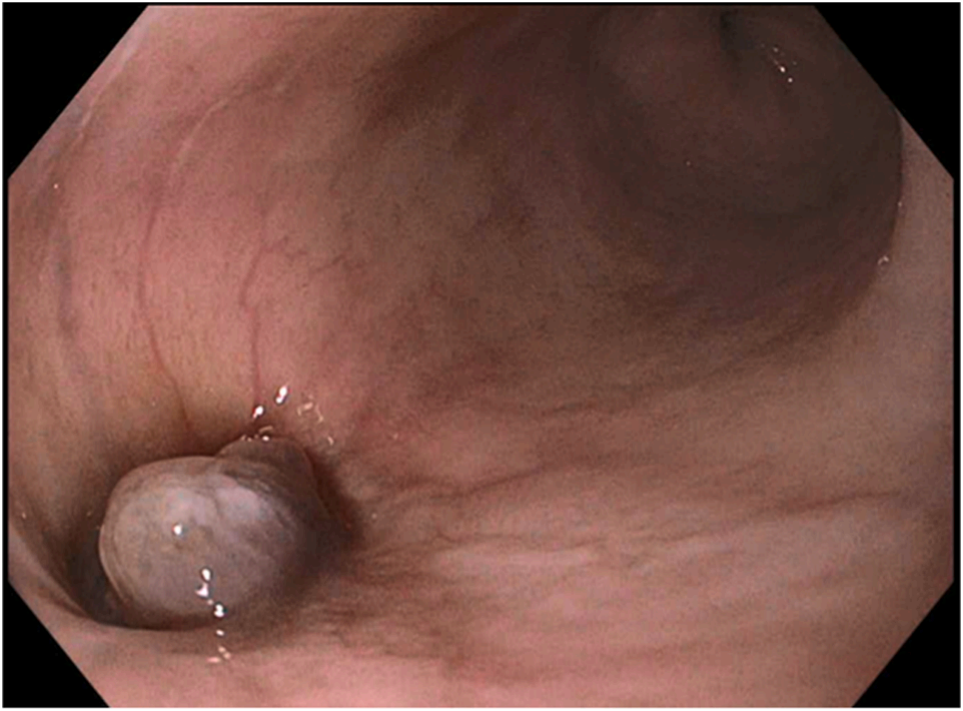
Endoscopy depicting the aortoesophageal fistula opening at the time of diagnosis.

In May 2022, the patient presented with fever, tachycardia, and elevated leukocytes and C-reactive protein and was diagnosed with a secondary AEF, which was confirmed using CT and esophagoscopy (Figures 1 and 2). All blood cultures were consistently negative.

**Figure 3:**
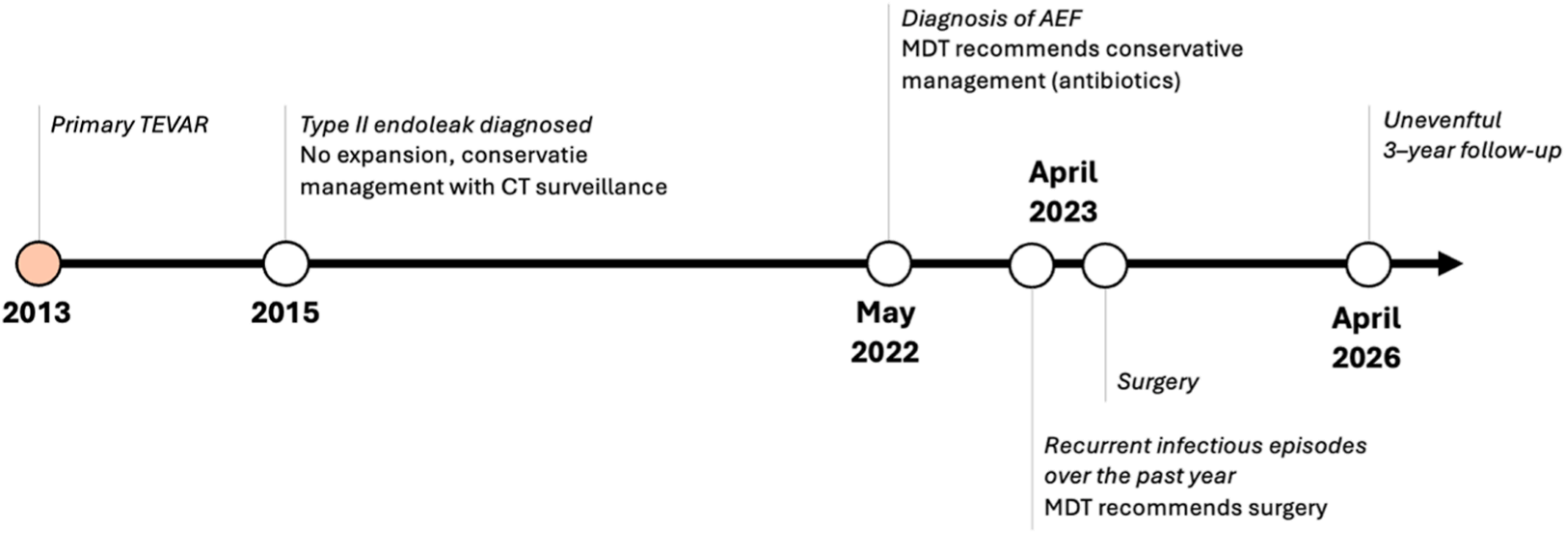
A timeline of the clinical course from the primary TEVAR to the last follow-up. *TEVAR*, Thoracic endovascular aortic repair; *CT*, computed tomography; *AEF*, aortoesophageal fistula; *MDT*, multidisciplinary team.

Given the operative risk, the multidisciplinary team recommended initial conservative management with amoxicillin-clavulanic acid (500 mg 3 times daily). However, this approach failed to achieve sustained clinical improvement, with the patient experiencing recurrent infectious episodes requiring multiple hospitalizations for intravenous broad-spectrum antibiotics. As a result, in April 2023, the case was again reviewed by the multidisciplinary team, and surgical removal of the infected stent graft and definitive aortic and esophageal repair was recommended. The patient was fully informed of the surgical risks, including paraplegia, renal failure, prolonged ventilatory support, and death. After extensive interdisciplinary discussions and informed consent, the patient opted for surgical treatment. Figure 3 depicts a timeline of the clinical course from the primary TEVAR to the last follow-up.

### Preoperative Evaluation

Routine preoperative assessment included transthoracic echocardiography, which demonstrated normal valvular function and preserved left ventricular ejection fraction. Coronary angiography revealed mild atherosclerosis without significant coronary artery stenosis.

### Surgical Procedure

**Figure 4:**
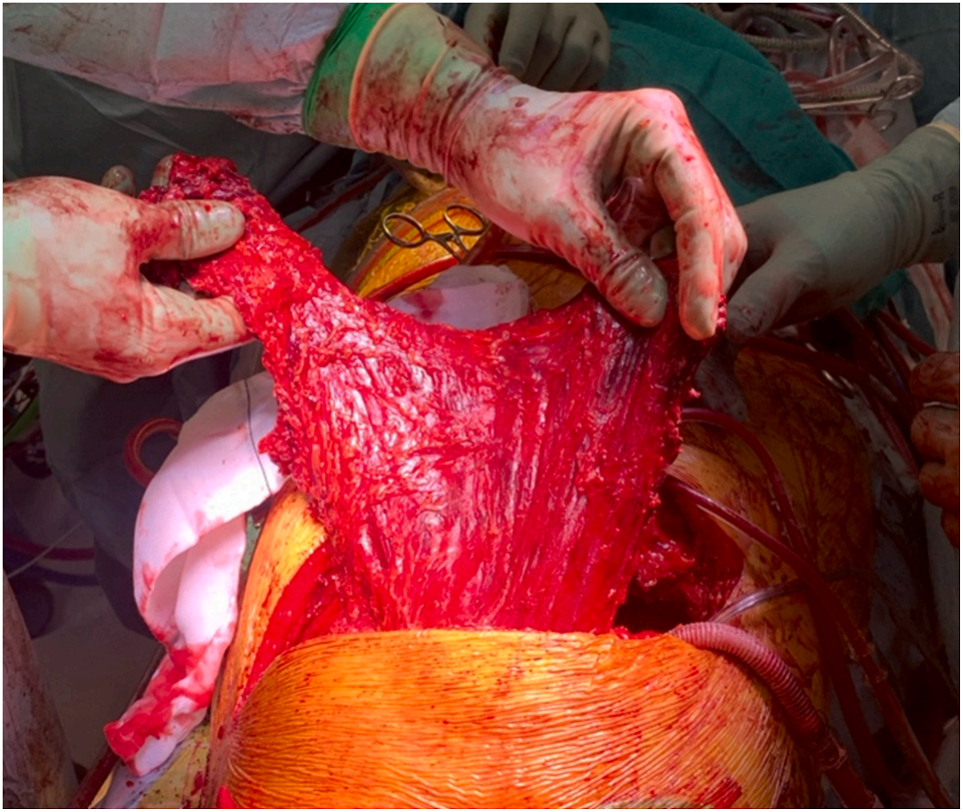
The fully mobilized latissimus dorsi.

The procedure was initiated by mobilizing the entire left latissimus dorsi muscle (Figure 4). A plastic reconstructive surgeon performed denervation while preserving the thoracodorsal vascular pedicle.

**Figure 5:**
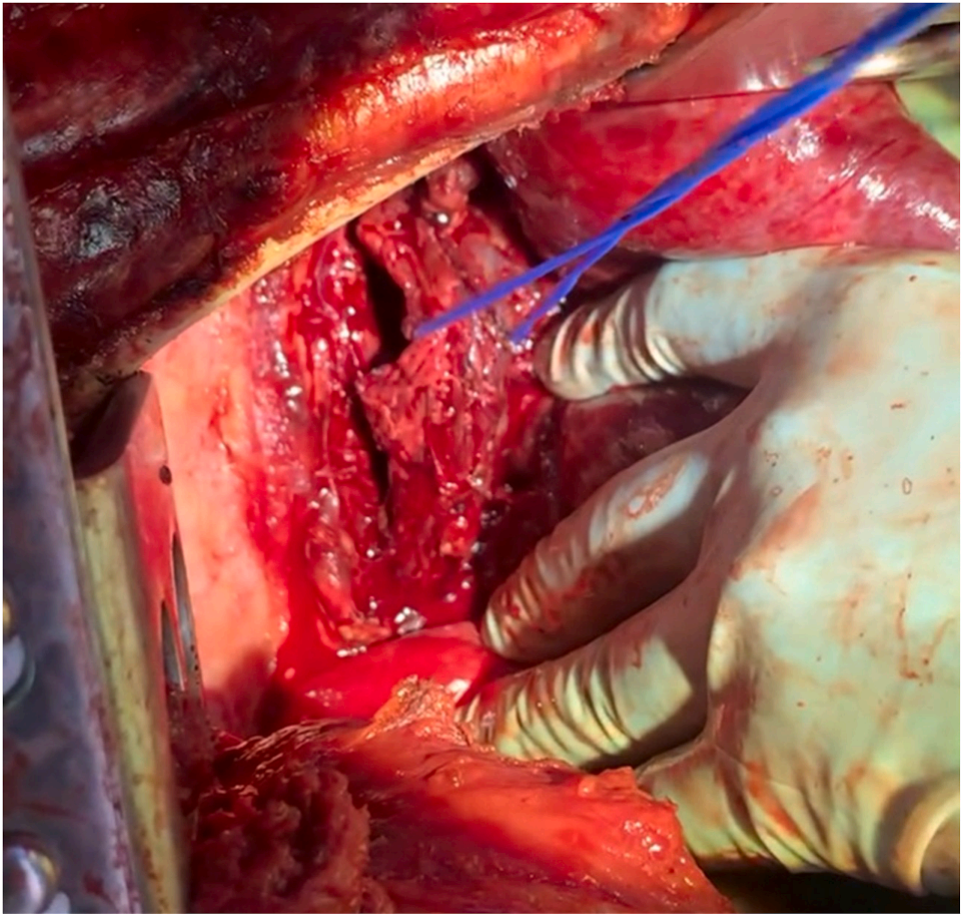
The aneurysm sac containing the infected stent graft was carefully mobilized.

The procedure was then advanced by the cardiothoracic surgeons through 2 thoracotomies in the fourth and sixth intercostal spaces, performed through a single skin incision. The lower segment of the aneurysm, which was densely adherent, was carefully dissected free (Figure 5). The stent graft was found to extend from the left subclavian artery to just above the diaphragm.

An incision was made in the right groin to expose the right femoral artery and vein, after which systemic heparinization was administered. Intravenous cannulation was performed, extracorporeal circulation (ECC) was initiated, and systemic hypothermia was induced. After reaching a core temperature of 18 °C, circulatory arrest was initiated.

The aneurysm sac was resected, and the proximal part of the stent graft was removed. A 24-mm Hemashield graft with a sidearm, presoaked in a solution of vancomycin 1 g and rifampicin 600 mg in 1000 mL of saline was anastomosed to the distal aortic arch. The proximal anastomosis was reinforced with an outer pericardial strip. Reperfusion was initiated with selective upper-body perfusion after clamping the aortic graft distally. Moderate systemic hypothermia was maintained to protect the spinal cord. Subsequently, the distal portion of the stent graft was resected, and the distal anastomosis was completed. Full circulation was then re-established, and the patient was systematically rewarmed.

The esophagus was then carefully mobilized by 2 esophageal surgeons, and the fistula was identified. Remnants of the aneurysmal sac were resected, and the fistulous tract was resected to fresh edges. The esophageal defect was closed with 2 running suture lines, and intraoperative esophagoscopy confirmed a tight closure. Extracorporeal circulation was discontinued, protamine was administered, and meticulous hemostasis was achieved.

The latissimus dorsi flap was transposed posteriorly into the upper thoracotomy field, fully interposed between the aortic graft and the esophagus, and secured to the esophagus with four interrupted sutures. Two large-bore drains were advanced into the left pleural cavity, and 2 subcutaneous drains were inserted into the dead space created by the latissimus dorsi mobilization.

**Figure 6:**
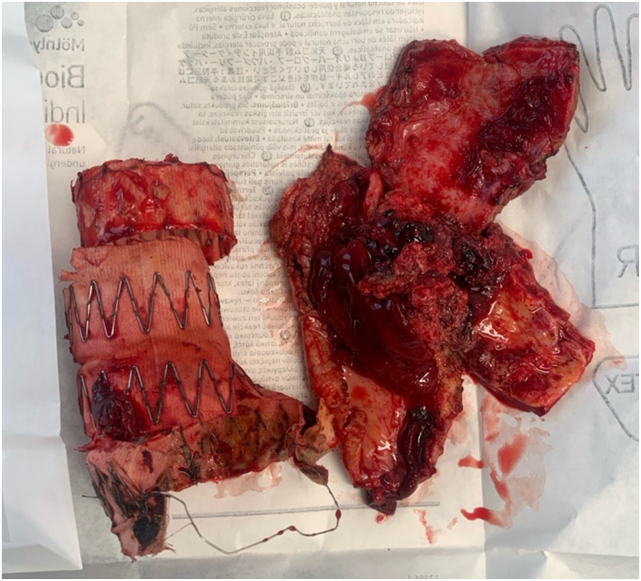
The infected stent graft together with parts of the aneurysm sac.

All resected infected material was sent for culturing and for bacterial detection using 16S ribosomal DNA sequencing (Figure 6). Additional ECC details included perfusion time: 224 minutes, aortic clamping time: 0 minutes, circulatory arrest: 21 minutes, and lowest body temperature: 18 °C.

During cooling, as anticipated, the patient developed hypothermia-induced ventricular fibrillation, which was managed expectantly, as is standard practice during deep hypothermia, with no immediate defibrillation required. A left ventricular vent was not used, because adequate decompression was achieved via ECC with vacuum-assisted venous drainage, and the heart remained decompressed throughout circulatory arrest. After completion of the proximal aortic anastomosis and initiation of selective upper-body perfusion, gradual rewarming was commenced. Defibrillation was performed once the core temperature exceeded 30 °C, successfully restoring sinus rhythm.

### Postoperative Course

The patient was kept on prolonged ventilatory support because of concerns regarding airway protection and was extubated on postoperative day (POD) 2. Postoperatively, the patient developed atrial fibrillation, which was managed conservatively, and acute kidney injury, which required temporary continuous renal-replacement therapy.

**Figure 7:**
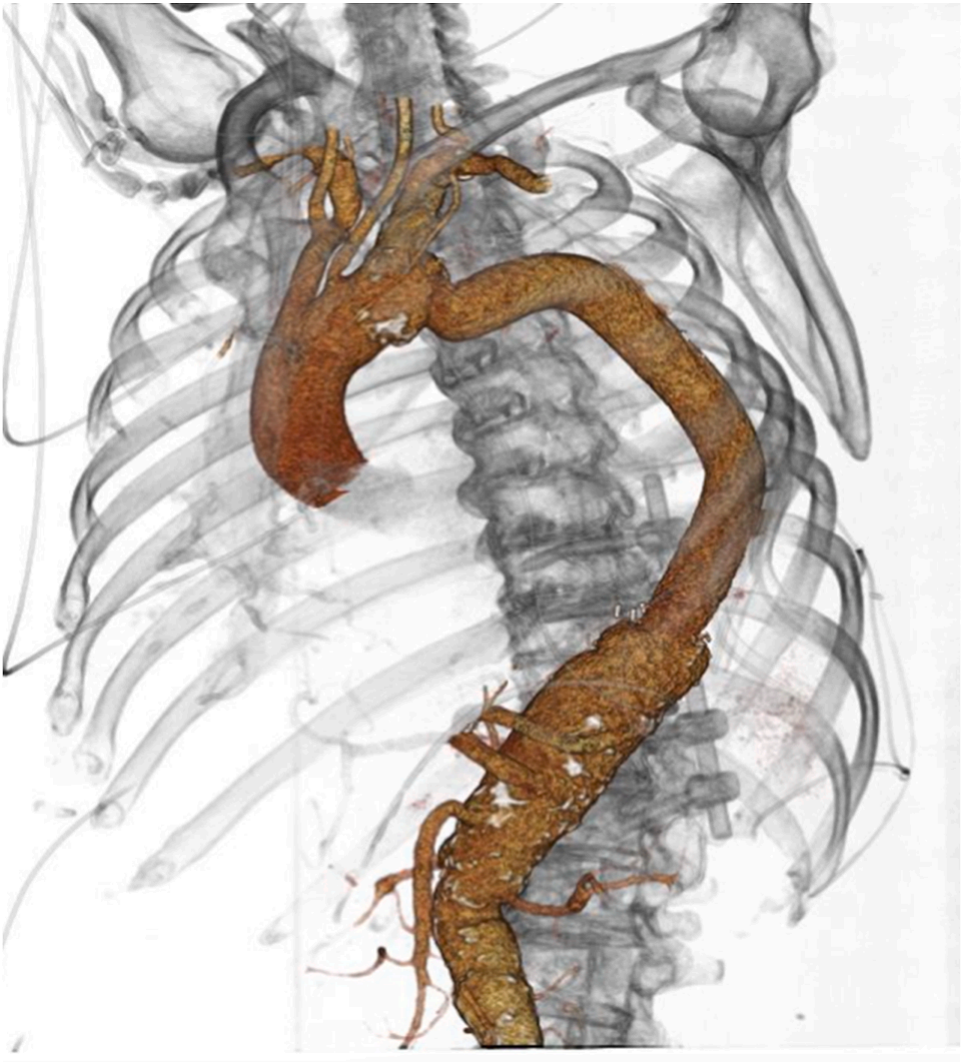
Three-dimensional reconstruction of the new aortic graft.

On POD 7, CT with oral contrast demonstrated no evidence of leakage or abscess formation. A liquid diet was initiated with gradual dietary advancement, and diuretic therapy was continued. Empiric antibiotic therapy with imipenem/cilastatin and vancomycin was started perioperatively and antifungal therapy with caspofungin was initiated postoperatively after the discovery of elevated beta-D-glucan levels. On POD 11, repeat CT esophagography and gastroscopy confirmed intact repair with no evidence of complications. On POD 13, the patient developed hoarseness, and an evaluation by an otolaryngologist identified suspected left recurrent laryngeal nerve palsy. Figure 7 depicts a 3-dimensional reconstruction of the aortic graft.

On POD 21, the patient was transferred to a local hospital, where continuous renal-replacement therapy was discontinued, and antibiotic therapy was continued pending finalization of microbiological results. The patient was clinically stable and functionally independent. He was discharged from the local hospital on May 17, 2023 (POD 30), with plans for elective otolaryngology follow-up.

### Follow-Up

The patient's general condition gradually improved, allowing a progressive return to daily activities. Renal function also improved, with laboratory values returning to preoperative levels.

Cultures obtained from the excised stent graft revealed growth of *Enterococcus faecium* and 2 different strains of *Staphylococcus epidermidis.* 16S ribosomal DNA analysis identified 2 anaerobic species, *Veillonella* spp., and *Prevotella melaninogenica*. Intravenous antimicrobial therapy with imipenem/cilastatin, vancomycin, and caspofungin was administered for 6 weeks under the supervision of a specialized graft infection team. Therapy was then sequentially tailored: imipenem/cilastatin was replaced by oral metronidazole, and vancomycin was replaced by dalbavancin under therapeutic drug monitoring. Subsequently, caspofungin was replaced by oral fluconazole. After 11 weeks, fluconazole was discontinued, and metronidazole was replaced by oral clindamycin due to adverse effects.

Fluorodeoxyglucose positron emission tomography (FDG-PET) performed 6 months postoperatively demonstrated normal graft uptake, and inflammatory parameters had normalized. At 18 months postoperatively, repeat FDG-PET confirmed persistently normal uptake with continued normalization of inflammatory markers, and the patient remained clinically well. Accordingly, all antimicrobial therapy was discontinued. A repeat FDG-PET performed shortly after discontinuation of antimicrobial therapy confirmed persistently low uptake. At 2-year follow-up, the patient had been off antimicrobial therapy for over 6 months, remained clinically well, hoarseness had fully resolved, and FDG-PET remained normal. At 3-year follow-up, the patient reported only occasional mild chest discomfort near the surgical scars. Lifelong surveillance through the infectious diseases clinic has been planned.

## Discussion

This case report illustrates the complexity of managing AEF as a rare but severe complication after TEVAR, particularly in the setting of infection refractory to conservative management. The successful surgical outcome was achieved through a single-stage approach, involving complete resection of the infected stent graft, in situ replacement with a new vascular graft, primary repair of the esophageal defect, and interposition of a pedicled latissimus dorsi muscle flap. Despite the surgical complexity, the postoperative course and follow-up were ultimately favorable. The authors emphasize the importance of stepwise de-escalation and eventual discontinuation of antimicrobial therapy under careful monitoring by an infectious disease specialist.

A key aspect of this case was the timely transition from conservative to definitive surgical management, driven by treatment failure and recurrent infectious episodes. This case also highlights the importance of remaining willing to reassess an initial conservative approach when clinical circumstances evolve. Given the lack of established guidelines and the limited number of reported cases, this transition required careful multidisciplinary evaluation.^1^^,^^14^ In addition, as the number of patients treated with TEVAR continues to increase, the incidence of secondary AEF is likely to increase as well, underscoring the relevance of this case as a meaningful contribution to the scarce literature on definitive management strategies.^1^^,^^3^

Several surgical strategies for the management of secondary AEF have been reported, most commonly involving staged procedures. Most involve initial esophageal diversion or esophagectomy, with immediate or delayed reconstruction, followed by definitive vascular repair.^9^, ^10^, ^11^ Reports describing single-stage surgical management of secondary AEF remain limited.^12^ The most recent report, describing 3 patients with AEF after TEVAR, demonstrated effective infection control with simultaneous aortic and esophageal repair in all 3 cases, thereby avoiding staged procedures. Tissue separation was achieved using an omental flap, in contrast to the latissimus dorsi flap used in the present case.^13^

In our case, the combined use of an antibiotic-soaked vascular graft and a pedicled latissimus dorsi muscle flap provides both antimicrobial protection and durable tissue separation between the aortic graft and the esophagus.^15^^,^^16^ The choice of a latissimus dorsi flap was based on the authors' experience and its well-established characteristics in contaminated fields.^17^^,^^18^ Compared with pleura and pericardium, the latissimus dorsi provides a well-vascularized, bulky, and robust flap with a wide arc of rotation, enabling effective mechanical separation between the aortic graft and the esophagus.^17^ Although the omentum is also well vascularized, it varies considerably in size and configuration, and its reach may be limited even when adequately pedicled on either gastroepiploic artery.^19^^,^^20^ Furthermore, its use requires an additional abdominal procedure, increasing operative complexity.^20^

Staged procedures inherently expose patients to multiple major operations, prolonged hospitalization, and cumulative perioperative risk.^12^ In contrast, a single-stage approach enables immediate eradication of the infectious source, definitive vascular reconstruction, and simultaneous restoration of esophageal integrity, avoiding the need for esophageal diversion, nutritional compromise, and delayed reconstruction, all of which are associated with significant morbidity and impaired quality of life.^21^ From both a logistical and patient-centered standpoint, single-stage repair may shorten the total treatment course and facilitate earlier return to normal function. However, this approach requires careful patient selection, meticulous surgical planning, and a highly experienced multidisciplinary team, as it concentrates the entire technical complexity into a single procedure.

In conclusion, the single-stage approach presented here yielded excellent outcomes with an uneventful clinical course at 3-year follow-up. These results underscore the importance of careful patient selection and close multidisciplinary collaboration, involving thoracic, vascular, esophageal, and plastic surgeons, anesthesiologists, intensivists, radiologists, nuclear medicine specialists, and infectious disease specialists.

## Conflict of Interest Statement

The authors reported no conflicts of interest.

The *Journal* policy requires editors and reviewers to disclose conflicts of interest and to decline handling or reviewing manuscripts for which they may have a conflict of interest. The editors and reviewers of this article have no conflicts of interest.
